# A cryoprotectant induces conformational change in glyceraldehyde-3-phosphate dehydrogenase

**DOI:** 10.1107/S2053230X18004557

**Published:** 2018-04-16

**Authors:** Yong Ju Kim

**Affiliations:** a Department of Herbal Medicine Resources, College of Environmental and Bioresource Sciences, Chonbuk National University, Iksan 54596, Republic of Korea; b Department of Lifestyle, College of Environmental and Bioresource Sciences, Chonbuk National University, Iksan 54596, Republic of Korea

**Keywords:** crystal structure, glyceraldehyde-3-phosphate dehydrogenase, GAPDH, cryoprotectants, trehalose

## Abstract

Glyceraldehyde-3-phosphate dehydrogenase (GAPDH), a glycolytic enzyme, catalyses the conversion of d-glyceraldehyde 3-phosphate to 1,3-bisphospho­glycerate. While mammalian and yeast GAPDHs are multifunctional proteins that have additional functions beyond those involved in glycolysis, including reactions related to nuclear RNA transport, DNA replication/repair, membrane fusion and cellular apoptosis, *Escherichia coli* GAPDH (*ec*GAPDH) has only been reported to function in glycolysis. The S-loop of GAPDH is required for interaction with its cofactor and with other proteins. In this study, the three-dimensional crystal structure of GAPDH treated with trehalose is reported at 2.0 Å resolution. Trehalose was used as a cryoprotectant for the GAPDH crystals. The structure of trehalose-bound *ec*GAPDH was compared with the structures of both NAD^+^-free and NAD^+^-bound *ec*GAPDH. At the S-loop, the bound trehalose in the GAPDH structure induces a 2.4° rotation compared with the NAD^+^-free *ec*GAPDH structure and a 3.1° rotation compared with the NAD^+^-bound *ec*GAPDH structure.

## Abstract 2

Trehalose is used in the cryoprotection of glyceraldehyde-3-phosphate dehydrogenase (GAPDH) crystals and induces conformational changes in GAPDH from *E. coli*. The conformational changes were independent of the duration of cryoprotectant soaking for up to 10 min.

## 1. Introduction   

Many crystals of biological macromolecules are sensitive to X-rays near room temperature and frequently suffer from radiation damage, especially when X-ray experiments are carried out on highly intense synchrotron beamlines (Hope, 1990 ▸). To prevent radiation damage from X-rays (Watenpaugh, 1991 ▸; Rodgers, 1994 ▸; Low *et al.*, 1966 ▸) and to facilitate the transport and simple storage of protein crystals, many cryoprotectants have been developed (Garman, 1999 ▸, 2003 ▸) and many cryoprotectant products are commercially available, such as CryoPro from Hampton Research.

In general, cryoprotectants are small polyols and organics, such as glycerol, ethylene glycol, 1,2-propanediol, diethylene glycol, 2-methyl-2,4-pentanediol, dimethyl sulfoxide or other nonvolatile alcohols (Pflugrath, 2015 ▸). Low-molecular-weight polyethylene glycols (PEGs), such as PEG 200 or PEG 400, are suitable cryoprotectants in many cases. Higher molecular-weight PEGs can also be used, albeit with more difficulty than lower molecular-weight PEGs. High concentrations of salts, such as lithium formate (Rubinson *et al.*, 2000 ▸), or carboxylic acids, such as malonate, can also be used (Holyoak *et al.*, 2003 ▸), as can sugars such as sucrose, trehalose, sorbitol, xylitol or glucose (Pflugrath, 2015 ▸).

Glyceraldehyde-3-phosphate dehydrogenase (GAPDH) is an enzyme that catalyses the conversion of glyceraldehyde 3-phosphate to 1,3-bisphosphoglycerate using NAD^+^ as a cofactor (Baker *et al.*, 2014 ▸) and is known to be a moonlighting protein (Savreux-Lenglet *et al.*, 2015 ▸). This enzyme plays multiple roles in the regulation of mRNA stability (Zhou *et al.*, 2008 ▸), intracellular membrane trafficking (Sirover, 2012 ▸), iron uptake and transport (Zaid *et al.*, 2009 ▸), DNA replication and repair (Zheng *et al.*, 2003 ▸), and nuclear RNA transport (Dastoor & Dreyer, 2001 ▸). In particular, yeast GAPDH is inhibited by trehalose (Araiza-Olivera *et al.*, 2010 ▸). An important region of GAPDH is the S-loop (residues 178–201), a long, winding region of the enzyme that is known to interact with some proteins (Kosova *et al.*, 2017 ▸; Duée *et al.*, 1996 ▸). The S-loop region of NAD^+^-free GAPDH has a very flexible shape and thus does not show clear electron density (Ferreira-da-Silva *et al.*, 2006 ▸; Querol-García *et al.*, 2017 ▸). On the other hand, in NAD^+^-bound GAPDH S-loop fixation occurs by the formation of a complex with the coenzyme NAD^+^ (Kitatani *et al.*, 2006 ▸).

GAPDHs have been isolated from bacteria, archaea, prokaryotes, eukaryotes, plants and mammals. Under normal cellular conditions, GAPDH primarily has a tetrameric conformation composed of four identical 35 kDa subunits (Nicholls *et al.*, 2012 ▸; Frayne *et al.*, 2009 ▸). GAPDH consists of an NAD^+^-binding domain (residues 2–148 and 312–330), which is composed of α/β dinucleotide-binding folds or Rossmann folds, and a catalytic domain (residues 149–311), which is composed of eight antiparallel β-sheets with an α-helix and several short loops (Duée *et al.*, 1996 ▸; Yun *et al.*, 2000 ▸). The activity of GAPDH requires the binding of NAD^+^.

In this study, the purification, crystallization and cryo­protection of *Escherichia coli* GAPDH (*ec*GAPDH) and the three-dimensional structure of trehalose-bound *ec*GAPDH are described. The GAPDH crystals were cryoprotected with a solution containing 15% of the disaccharide trehalose.

## 2. Materials and methods   

### 2.1. Preparation of GAPDH   

*E. coli* BL21 (DE3) cells were grown on LB agar medium containing 100 µ*M* ampicillin. A single colony was cultivated in 100 ml LB medium overnight at 37°C. The next day, a main culture was cultivated in 1 l LB medium for 4 h at 37°C. The cultured cells were harvested by centrifugation (4°C, 8000 rev min^−1^, 5 min). The harvested cells were resuspended in buffer *A* (20 m*M* Tris, 130 m*M* NaCl pH 7.5). The cells were disrupted by sonication on ice and the cell lysate was then separated from the cell debris by centrifugation (4°C, 20 000 rev min^−1^, 20 min). The supernatant was loaded onto a HisTrap column (5 ml; GE Healthcare, Little Chalfont, England) that had been equilibrated with buffer *A*. The column was washed with buffer *A* and the proteins bound to the HisTrap column were eluted with buffer *B* (20 m*M* Tris, 130 m*M* NaCl, 0.2 *M* imidazole pH 7.5). The extracted active proteins, including *ec*GAPDH, were loaded onto a HiTrap heparin column (5 ml; GE Healthcare) equilibrated with buffer *A* (Reisz *et al.*, 2016 ▸). After washing the column with buffer *A*, buffer *C* (20 m*M* Tris pH 7.5, 1 *M* NaCl) was gradually applied as an elution buffer. *ec*GAPDH was eluted at concentrations of 200–300 m*M* NaCl and was dialysed thoroughly against buffer *A*.

### 2.2. Crystallization   

Purified *ec*GAPDH was concentrated to 20 mg ml^−1^ using Amicon filters (30 kDa cutoff; Sigma–Aldrich, St Louis, Missouri, USA). Concentrated *ec*GAPDH (2 µl) was mixed with reservoir solution (2 µl) consisting of 2.8 *M* ammonium sulfate, 0.1 *M* 2-(*N*-morpholino)ethanesulfonic acid (MES) pH 5.5–6.5 at 4°C. GAPDH crystals grew within two weeks using the hanging-drop method. X-ray data were collected from GAPDH crystals treated with a cryoprotectant consisting of 15%(*v*/*v*) trehalose at 100 K. Crystallization information is given in Table 1 ▸.

### 2.3. Data collection and processing   

X-ray diffraction data were collected on beamline 7A at the Pohang Accelerator Laboratory (PAL 7A SB I; Pohang, Republic of Korea) using a CCD detector (ADSC Quantum 270) at an X-ray wavelength of 1.0 Å. All images were indexed, integrated and scaled using the *XDS* program package and the *CCP*4 program *SCALA*. The crystals belonged to the tetragonal space group *I*4_1_22, with unit-cell parameters *a* = 121.29, *b* = 121.29, *c* = 156.06 Å, α = 90, β = 90, γ = 90°. Data-collection and processing statistics are given in Table 2 ▸.

## 3. Structure determination and refinement   

Initial phases for GAPDH were obtained by a conventional molecular-replacement protocol (rotation, translation and rigid-body fitting) using the structure of *E. coli* GAPDH (PDB entry 1s7c; Berkeley Structural Genomics Center, unpublished work) as an initial search model. The model was fitted more appropriately by simulated annealing in *CNS*. Manual rebuilding of the GAPDH model and electron-density interpretation were performed after each refinement cycle using *Coot* (Emsley *et al.*, 2010 ▸). Restrained, individual *B* factors were refined and the crystal structure was finalized using the *CCP*4 program *REFMAC*5 and other programs in the *CCP*4 suite (Winn *et al.*, 2011 ▸). GAPDH was modelled with TLS refinement using anisotropic temperature factors for all atoms. The final model had *R*_free_ and *R*_cryst_ factors of 20.5% and 18.4%, respectively (Table 3 ▸). Structure validation was performed with *SFCHECK* (Vaguine *et al.*, 1999 ▸), *PROCHECK* (Laskowski *et al.*, 1996 ▸) and *ADIT* (Bhat *et al.*, 2001 ▸). *DynDom*, a program that determines domain movement and relative inter-domain rotation angles in proteins for which two conformations are available, was used to study domain rotation (Poornam *et al.*, 2009 ▸; Hayward *et al.*, 1997 ▸). Coordinates have been deposited in the Protein Data Bank with accession code 5za0.

## 4. Results and discussion   

**Figure 1:**
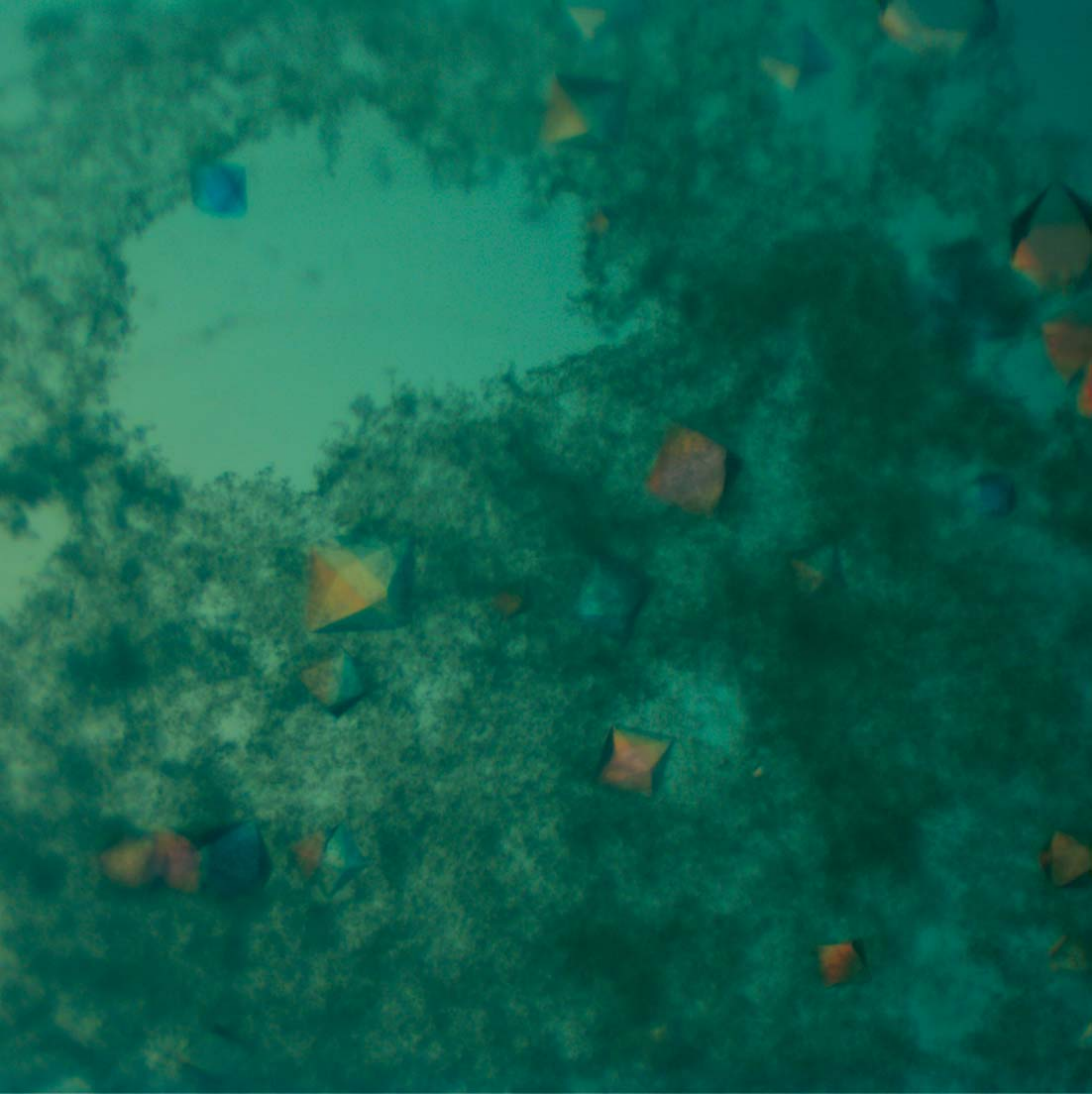
Crystals of *ec*GAPDH.

GAPDH was purified from *E. coli* without any genetic manipulation. Despite the use of a nonrecombinant gene, GAPDH could surprisingly be purified using a HisTrap column. Approximately 2 mg of purified *ec*GAPDH was obtained from a 1 l culture. For crystallization purposes, *ec*GAPDH was concentrated to approximately 20 mg ml^−1^ using an Amicon concentrator (30 kDa cutoff; Sigma–Aldrich). Although *ec*GAPDH was stable at room temperature, crystals suitable for X-ray analysis were grown by the hanging-drop method at 4°C (Fig. 1 ▸). Tetragonal bipyramidal crystals appeared within two weeks and grew for a further week. For data collection, the crystals were cryoprotected with 15% trehalose. In the X-ray studies, a single crystal of *ec*GAPDH was mounted in a cryoloop. The crystals showed significant overloads. Thus, even when the resolution was reduced, X-ray diffraction was measured at 80% attenuation. Nevertheless, these crystals diffracted to 2.0 Å resolution on PAL 7A SB I (Supplementary Fig. S1).

The structure was solved by molecular replacement using the *E. coli* GAPDH structure (PDB entry 1s7c) as a starting model. Molecular replacement was performed using *Phaser* from the *CCP*4 suite. The final *ec*GAPDH model had *R*_free_ and *R*_cryst_ factors of 20.5% and 18.4%, respectively. The solved structure had a monomer in the asymmetric unit. The monomer contained 330 residues, one sulfate ion, one trehalose molecule and 171 water molecules. The number of water molecules in the current GAPDH structure is restricted despite the high resolution and low mosaicity. To study the conformational changes induced by the binding of trehalose to *ec*GAPDH, a detailed structural comparison of trehalose-free and trehalose-bound GAPDH was performed.

A list of commonly used sugar cryoprotectants is shown in Supplementary Table S1. Among them, disaccharides such as sucrose, maltose and trehalose have been used to cryoprotect GAPDH crystals. Glycerol, a commonly used cryoprotectant, has also been used in a comparison with sugar-protected GAPDH. Mixtures of 30% glycerol and 15% sugars were used for cryoprotection. None of the resulting crystals exhibited any signs of cracking or melting during treatment with the cryoprotectants. The crystals obtained using glycerol as a cryoprotectant showed a resolution of 2.1 Å. The crystals obtained using sucrose and maltose showed resolutions of 2.4 and 2.2 Å, respectively. The crystals obtained using trehalose showed a resolution of 2.0 Å. Although sucrose and maltose have similar structures to that of trehalose, these sugars did not bind to *ec*GAPDH and glycerol was not found, even though glycerol has previously been reported to be bound in GAPDH structures (Mukherjee *et al.*, 2010 ▸; Cook *et al.*, 2009 ▸; Robien *et al.*, 2006 ▸; Chaikuad *et al.*, 2011 ▸; Querol-García *et al.*, 2017 ▸; Moreau *et al.*, 2017 ▸). Only the GAPDH structure with trehalose was significantly different from published structures.

**Figure 2:**
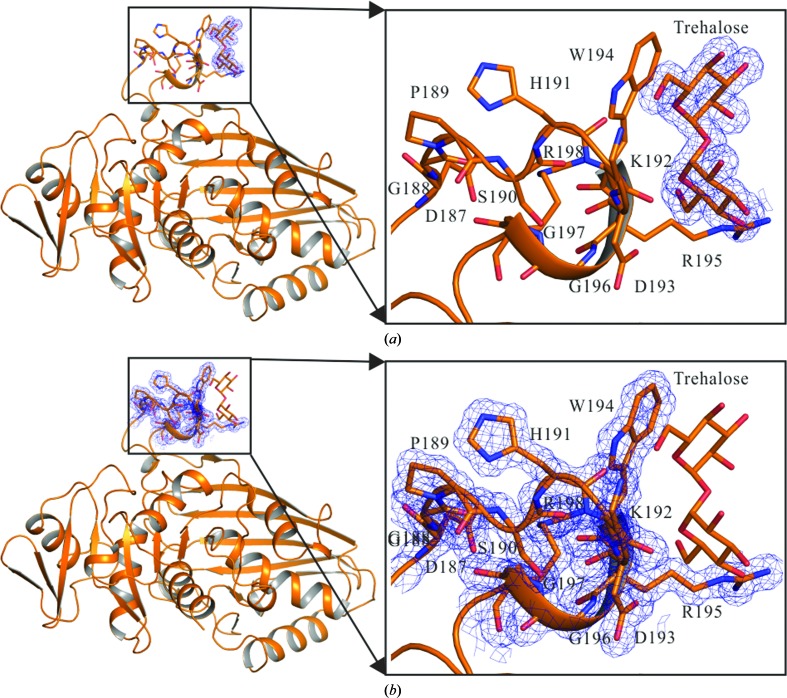
Electron-density map of trehalose and the *ec*GAPDH S-loop. *ec*GAPDH is shown as a cartoon model with trehalose and S-loop residues shown as orange stick models. (*a*) 2*F*_o_ − *F*_c_ electron-density map of trehalose contoured at 3.0σ (blue mesh). (*b*) 2*F*_o_ − *F*_c_ electron-density map of the S-loop residues contoured at 3.0σ (blue mesh).

**Figure 3:**
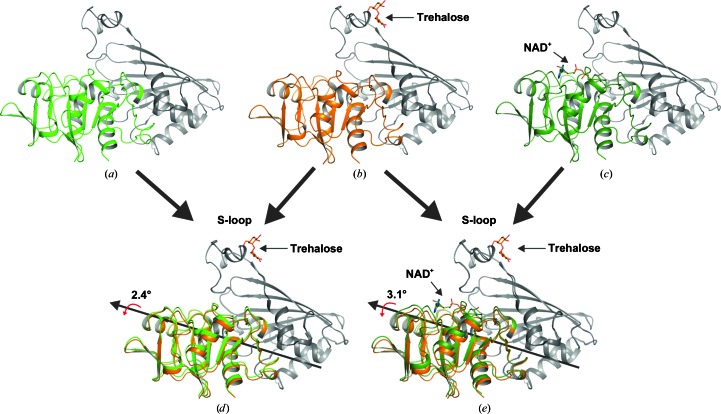
Rotation of domains in trehalose-bound *ec*GAPDH compared with NAD^+^-free *ec*GAPDH and NAD^+^-bound *ec*GAPDH. The fixed domain is represented in black. (*a*) NAD^+^-free *ec*GAPDH (PDB entry 1dc5; green). (*b*) Trehalose-bound *ec*GAPDH (orange). (*c*) NAD^+^-bound *ec*GAPDH (PDB entry 1dc6; forest). (*d*) The calculated relative rotation angle of trehalose-bound *ec*GAPDH compared with NAD^+^-free *ec*GAPDH is 2.4°. (*e*) The calculated relative rotation angle of trehalose-bound *ec*GAPDH compared with NAD^+^-bound *ec*GAPDH is 3.1°.

An electron-density map clearly indicated ordered tre­halose molecules bound to the protein. One trehalose molecule bound to *ec*GAPDH in the S-loop region (Fig. 2 ▸). As described above, the S-loop region of GAPDH without NAD^+^ bound as a cofactor is very flexible and does not exhibit definite electron density (Querol-García *et al.*, 2017 ▸). Despite the absence of NAD^+^, trehalose stabilized the S-loop of the enzyme. As a result, the electron-density map of the S-loop region of the protein was clear. The rotation angle and the relative rotation angle between the NAD^+^-binding domain and the catalytic domain were measured. The GAPDH molecule shows a rotation of approximately 2.4–3.1° when superpositioned on the NAD^+^-free *ec*GAPDH (PDB entry 1dc5) and/or the NAD^+^-bound *ec*GAPDH (PDB entry 1dc6) structures (Yun *et al.*, 2000 ▸; Fig. 3 ▸). The inhibition of yeast GAPDH is reportedly proportional to the concentration of trehalose (Araiza-Olivera *et al.*, 2010 ▸). The GAPDHs from yeast and *E. coli* have similar structures. Although *ec*GAPDH has not been reported to be inhibited by trehalose, trehalose induced a conformational change in *ec*GAPDH in the current structure. The rotation of GAPDH also induced a conformational change in its active site. This suggests that the binding of trehalose to GAPDH induced a conformational change in its active site to prevent the binding of NAD^+^, although the NAD^+^- and trehalose-binding sites differ from one another.

To examine the time dependence of the cryoprotectant effect on GAPDH crystals, the cryoprotectant soaking time was varied from 1 to 10 min and the data for all crystals from each time were evaluated systematically. The GAPDH crystals neither cracked nor melted as a result of the penetration of cryoprotectants into the solvent channels. These results suggested that the conformational changes in GAPDH owing to the binding of trehalose were independent of soaking time for up to 10 min.

## Supplementary Material

PDB reference: glyceraldehyde-3-phosphate dehydrogenase, 5za0

Supplementary Table S1 and Supplementary Figure S1.. DOI: 10.1107/S2053230X18004557/ek5003sup1.pdf

## Figures and Tables

**Table 1:** Crystallization conditions

Method	Hanging-drop vapour diffusion
Plate type	24-well plates
Temperature (°C)	4
Protein concentration (mg ml^−1^)	20
Buffer composition of protein solution	20 m*M* Tris pH 7.5, 0.1 *M* NaCl
Composition of reservoir solution	0.1 *M* MES pH 5.5–6.5, 2.8 *M* ammonium sulfate
Volume and ratio of drop	2 µl:2 µl
Volume of reservoir (ml)	1

**Table 2:** Data collection and processing Values in parentheses are for the outer shell.

Diffraction source	PAL 7A SB I
Wavelength (Å)	1.00
Temperature (K)	100
Detector	ADSC Quantum 270
Crystal-to-detector distance (mm)	230
Rotation range per image (°)	1
Total rotation range (°)	90
Exposure time per image (s)	1
Space group	*I*4_1_22
*a*, *b*, *c* (Å)	121.29, 121.29, 156.06
α, β, γ (°)	90, 90, 90
Mosaicity (°)	0.126
Resolution range (Å)	47.88–2.00 (2.10–2.00)
Total No. of reflections	284143 (41653)
No. of unique reflections	39522 (5706)
Completeness (%)	99.6 (99.7)
Multiplicity	7.2 (7.3)
〈*I*/σ(*I*)〉	21.8 (8.1)
*R*_meas_	0.061 (0.257)
Overall *B* factor from Wilson plot (Å^2^)	22.9

**Table 3:** Structure solution and refinement

Resolution range (Å)	47.7–2.00
Completeness (%)	99.5
No. of reflections	37461
Final *R*_cryst_ (%)	18.4
Final *R*_free_ (%)	20.5
No. of atoms/residues	
Protein	2451/330
Others	199/173
Sulfate	5/1
Trehalose	23/1
Water	171/171
Total	2650/503
R.m.s. deviations	
Bond lengths (Å)	0.010
Bond angles (°)	1.284
Ramachandran plot	
Most favoured (%)	97
Allowed (%)	2.7
Disallowed (%)	0.3
