# Supplementary material for: A cryoprotectant induces conformational change in glyceraldehyde-3-phosphate dehydrogenase
Source: Acta Crystallogr F Struct Biol Commun. 2018 Apr 16;74(Pt 5):277–82. doi: 10.1107/S2053230X18004557 (PMC5931139; doi:10.1107/S2053230X18004557)
Supplement: Supplementary file 1 [file f-74-00277-sup1.pdf]

Acta Cryst F

STRUCTURAL BIOLOGY
COMMUNICATIONS

Volume 74 (2018)

Supporting information for article:

A cryoprotectant induces conformational change in glyceraldehyde-3-phosphate dehydrogenase

Yong Ju Kim

Table S1. List of sugar cryoprotectants (CryoPro from Hampton Research)

Tube No.	Cryoprotectant	Mr	Formula	Structure (National institute of standards and technology)
25	Sucrose	342.30	C12H22O11	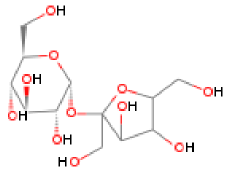
26	D-Sorbitol	182.17	C6H14O6	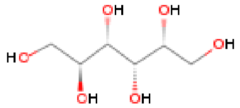
27	D-(+)-Maltose	342.30	C12H22O11	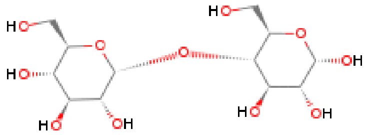
28	Meso-Erythritol	122.12	C4H10O4	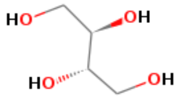
29	Xylitol	152.15	C5H12O5	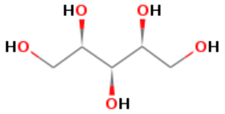
30	myo-Inositol	180.16	C6H12O6	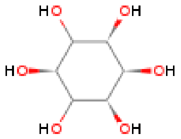
31	D-(+)-Raffinose	504.44	C18H32O16	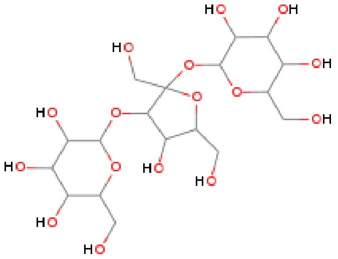
32	Trehalose	342.30	C12H22O11	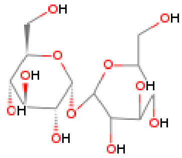
33	D-(+)-Glucose	180.16	C6H12O6	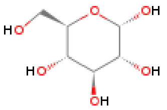

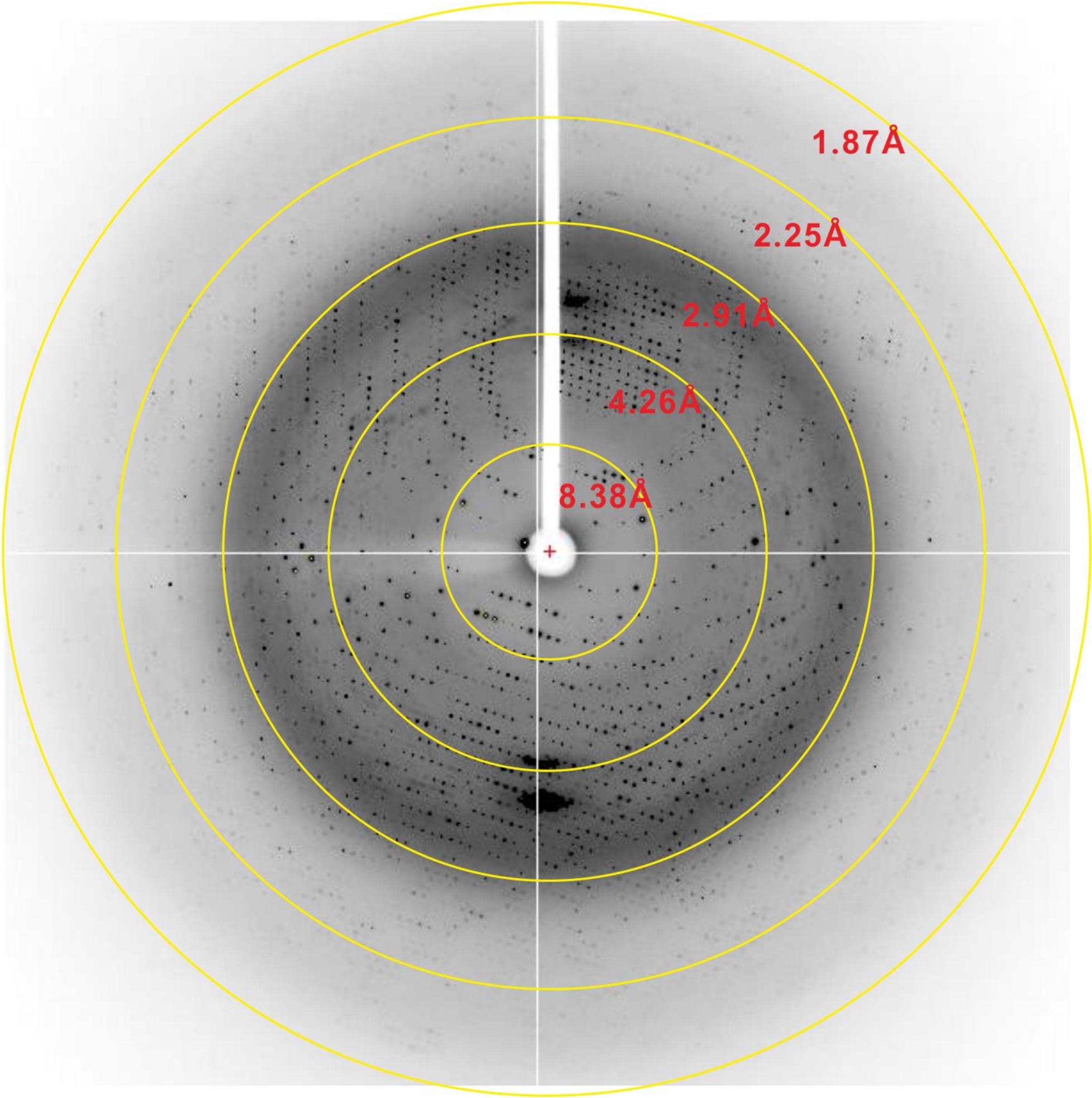

Figure S1 X-ray diffraction pattern of an ecGAPDH crystal recorded on PAL 7A SB I.
